# Limitation-circumventing and strength-capitalizing hydrogel potentiates durable antitumor immunity and robust abscopal effect in radiotherapy

**DOI:** 10.7150/thno.118974

**Published:** 2026-01-01

**Authors:** Haijun Li, Xianzhou Huang, Jin Yang, Liping Bai, Meiling Shen, Yaqin Zhao, Changyang Gong, Yanjie You, Qinjie Wu

**Affiliations:** 1 Department of Biotherapy, Cancer Center and State Key Laboratory of Biotherapy, West China Hospital, Sichuan University, Chengdu, 610041, P. R. China.; 2 Department 4 of Oncology, Cancer Center, The Second People's Hospital of Neijiang, Neijaing, 641000, P. R. China.; 3 Abdominal Oncology Ward, Cancer Center, West China Hospital, Sichuan University, Chengdu, 610041, P. R. China.; 4 State Key Laboratory of Biological Therapy, West China Hospital, Sichuan University, Chengdu, 610041, P. R. China.; 5 Department of Gastroenterology, People's Hospital of Ningxia Hui Autonomous Region, Ningxia Medical University, Yinchuan, 750002, P. R. China.; 6 Department of Gastroenterology, the Third Clinical Medical College of Ningxia Medical University, Yinchuan, 750002, P. R. China.

**Keywords:** hydrogel, radiotherapy, hypoxia, abscopal effect, immune memory

## Abstract

**Background:** Radiotherapy (RT)-induced antitumor immunity has attracted extensive attention. However, such antitumor immunity often proves inadequate to combat metastatic and recurrent tumors in clinical practice. While hypoxia severely limits the initiation of adequate systemic antitumor immune responses, the strength of such responses is further compromised by the blockage of effector T cells, ultimately undermining the ability of RT to elicit a robust abscopal effect and durable immune memory.

**Methods:** Hence, a limitation-circumventing strength-capitalizing hydrogel (UP) is developed to increase the efficacy of RT for a robust abscopal effect and durable antitumor immunity to cope with metastatic and recurrent tumors.

**Results:** With single-administration and radiation treatments, UP efficiently generates oxygen *in situ* to circumvent the limitation of RT hypoxia. After such limitations are overcome, the tumor-killing capacity of RT is significantly promoted, resulting in strong antitumor immune responses. Moreover, UP further inhibits immune checkpoints to reinvigorate effector T cells, capitalizing on the strength of RT-induced antitumor immune responses. With such strength-capitalization, RT-induced transient immune responses are amplified to systemic immunity, triggering a robust abscopal effect to eliminate distant metastasis and establishing durable immune memory against recurrence.

**Conclusions:** Consequently, UP potentiates the antitumor immunity of RT through circumventing the hypoxia barrier and capitalizing on immune activation. Our study provides new insights into RT enhancement.

## Introduction

As a crucial antitumor approach, radiotherapy (RT) is required for approximately 60%-70% of cancer patients 1,2. Apart from its effective control of local tumors, RT is frequently mentioned for its ability to induce systemic antitumor immunity, which has the potential to generate an abscopal effect to eliminate distant metastasis and immune memory to indirectly curb tumor recurrence 3,4. However, in clinical practice, the RT-induced antitumor immunity often proves inadequate for generating effective abscopal effects to combat metastatic and recurrent tumors 5-7. Limitations such as the inherent hypoxia of tumors severely attenuate the ability of RT to trigger a strong systemic antitumor immune response, while the strength of the RT-induced immune response is further compromised by inhibition of the tumor environment 8-12. Therefore, new strategies oriented at such limitations and strengths will provide new insights into enhancing the antitumor immunity of RT.

To augment RT-induced antitumor immunity, limitations must be overcome. Hypoxia, as a critical and pervasive limitation, is an important cause of the failure of RT to induce effective systemic antitumor immunity 13-16. Hypoxia is generally generated in tumors larger than 1 mm because of aberrant blood vessel development in solid tumors such as triple-negative breast cancer (TNBC), making RT nearly inevitable 17-20. Tumor hypoxia can severely limit tumor cell damage induced by RT, leading to the triggering of inadequate antitumor immune responses 10,21,22. Such inadequate antitumor immune responses do not generate effective abscopal effects or immune memory to cope with metastatic and recurrent tumors. Hence, circumventing the limitation of tumor hypoxia may efficiently improve the induction of strong antitumor immune responses by RT.

Furthermore, to promote the efficacy of the antitumor immune responses, the strength of such responses should be capitalized as well. In the tumor microenvironment, the strength of antitumor immune responses can be compromised by inhibitory factors, especially inhibitory factors targeting effector T cells 23-26. Programmed cell death protein-1 (PD-1) and programmed death-ligand 1 (PD-L1) are typical and critical inhibitory factors that can block the antitumor function of activated T cells. Fortunately, approaches targeting these factors have been developed 24-29. Thus, capitalizing on the strength of effector T cells with anti-PD-1/L1 antibodies (αPD-1/L1) may further promote the antitumor efficacy of RT-induced antitumor immune responses.

**Figure 1:**
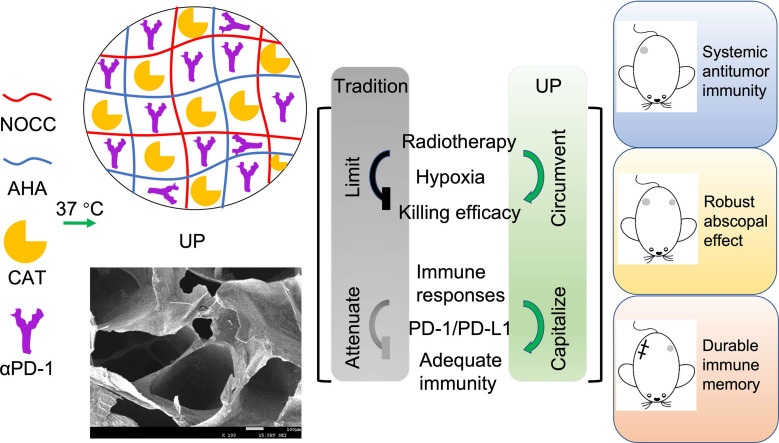
Illustration of the limitation-circumventing strength-capitalizing strategy of UP. The prepared injectable hydrogel UP potentiates adequate systemic antitumor immunity in RT through circumventing the hypoxia barrier and capitalizing on immune activation to trigger robust abscopal effects to eliminate distant metastasis and establish durable immune memory against recurrence.

Here, we developed a limitation-circumventing and strength-capitalizing hydrogel (UP) to increase the antitumor efficacy of RT for robust abscopal effects and durable antitumor immunity to cope with the metastasis and recurrence of TNBC (Figure 1). With one administration and radiation, UP, on the one hand, could efficiently catalyze and generate oxygen *in situ* to circumvent the limitation of hypoxia for RT 30,31. After overcoming this limitation, the killing effect of RT on tumor cells significantly improved, resulting in strong antitumor immune responses. On the other hand, UP could further relieve the effects of PD-1 and PD-L1 on the function of effector T cells, capitalizing on the strength of RT-induced antitumor immune responses. With such strength-capitalization, the induced transient immune responses amplify systemic immunity. Robust abscopal effects are triggered to eliminate distant metastasis, and robust immune memory is generated to curb recurrent tumors. Consequently, UP could potentiate the antitumor immune efficacy of RT on TNBC through circumventing the hypoxia barrier and capitalizing on immune activation, providing new insights into TNBC treatment and RT enhancement.

## Materials and Methods

### Materials

Hyaluronic acid (HA), catalase (CAT), N,O-carboxymethyl chitosan (NOCC), were all brought from Sigma Aldrich Chemical Co., USA. αPD-1 and other antibodies were from BioLegend, USA. Enzyme-linked immunosorbent assay (ELISA) kit of IFN-γ was from Invitrogen Co., USA. Dimethyl sulfoxide (DMSO), hydroxylamine hydrochloride (NH_2_OH·HCl), ammonium molybdate, sodium periodate (NaIO_4_), ethylene glycol, and Proteinase K were from Kelong Chemical Co., China. 4T1 cells and NIH-3T3 (3T3) cells were from the American Type Culture Collection (ATCC). BALB/c mice were from HuaFuKang Biotechnology Co., China. Animal experiments were approved by the Institutional Animal Care and Use Committee at West China Hospital, Sichuan University.

### Synthesis of aldehyaluronic acid (AHA)

AHA was synthesized through the oxidation of HA by NaIO_4_ 32. In brief, NaIO_4_ was added into 100 mL of HA solution in a round bottom flask. NaIO_4_:HA molar ratio was 2:5. The mixed solution was stirred at room temperature under light-proof conditions, 2 h later, adding ethylene glycol. The stir continued for 1 h to quench the reaction. The mixture was then purified through dialysis (MWCO = 3500) against double distilled water which was replaced every 8 h for 3 days. The product was next collected and further freeze-dried. The AHA oxidation degree (OD) was measured by the aldehyde group number through NH_2_OH·HCl method. NH_2_OH·HCl reacted with the aldehyde groups of AHA, generating oxime and HCl 32. The OD of AHA could be determined with the following equation: OD (%) = Mw × C_NaOH_ × (V_AHA_ - V_blank_)/(2 × m_AHA_) × 100%, where the molecular weight (Mw) of HA was 400 g/mol; C_NaOH_ was 0.1 mol/L; V_AHA_ was the consumed NaOH volume (mL) with AHA; V_blank_ was the consumed NaOH volume (mL) without AHA; and m_AHA_ was the mass of AHA (mg). ^1^H-NMR and Fourier transform infrared (FTIR) spectroscopy of HA and AHA was further performed.

### Preparation of UP

Briefly, 30 mg/mL NOCC and 30 mg/mL AHA from different NaIO_4_:HA molar ratios (2:5) in saline were mixed (1:1, v/v) at 4 °C. The hydrogels were then removed to 37 °C. The gelation times of the hydrogels were recorded. Afterward, when NOCC and AHA were mixed, αPD-1 and CAT were added to prepare UP. Rheological measurements of prepared hydrogels were performed at 37 °C in oscillatory mode via a HAAKE MARS RS6000 rheometer, Thermo Scientific, Germany 32. The gap size of upper plate was 1 mm. The storage modulus (G') and loss modulus (G'') were recorded. The hydrogels were next freeze-dried and observed by scanning electron microscopy (SEM), JSM-5900LV, JEOL, Japan.

### Analysis of UP-catalyzed oxygen generation

The generated oxygen was analyzed through an oxygen meter, JPBJ-608, INESA Scientific Instrument Co., Ltd., China. Before the measurement, ultrasonication of 30 min was performed to remove the dissolved oxygen in all buffer. First, to determine the optimal concentration of CAT in UP, UPs with different CAT concentrations (5 mg/L, 10 mg/L, 20 mg/L, 40 mg/L, 80 mg/L, and 160 mg/L) were prepared and added to 1 mL of 1 mmol/L H_2_O_2_ solution. The generated oxygen was detected and analyzed. To compare the oxygen generation ability, UP with the preferred CAT concentration and free CAT at the corresponding concentration were subsequently added to a 1 mL 1 mmol/L H_2_O_2_ solution. The generated oxygen was measured and recorded for 600 s. All the experiments were performed in triplicate.

### Catalytic activity of UP

The catalytic activity was determined by colorimetry. Briefly, 20 μL of 0.5 mg/mL Proteinase K was mixed with UP and free CAT at 37 °C. After certain time points (0 min, 15 min, 30 min, 60 min, 120 min, 240 min, and 480 min), the mixtures were added to the H_2_O_2_ solutions. One minute later, 0.5 mL of ammonium molybdate solution was added to terminate the reactions. The color of the solutions then changed from colorless to yellow. Next, the solution absorbance was detected at 400 nm, and the catalytic activities were calculated. All experiments were performed in triplicate.

### Drug release behavior *in vitro*

The drug release behavior of the NOCC-AHA hydrogels was evaluated by dialysis method. Before analysis, UP(BSA) was prepared to simulate the release behavior of protein from the NOCC-AHA hydrogel. Afterward, UP(BSA) and free BSA (with BSA mass of 70 mg) were put into separated dialysis bags with MWCO of 500-1000, respectively. The bags were next immersed in 10 mL of PBS with pH of 7.4 and 0.5 wt% Tween 80. All PBS samples were collected at each time intervals and replaced with fresh ones. The BSA concentration in collected samples were tested. All experiments were performed in triplicate.

### *In vitro* cell viability

The extracts of the hydrogels in DMEM or 1640 medium supplemented with 10% FBS were filtered through 0.22 μm sterile filters. Afterward, sequential dilutions were carried out (0%, 6.25%, 12.5%, 25%, 50%, and 100% of the extracts), which were added to the 96-well plates with pre-seeded cells and incubated with the cells for 24 h or 48 h. Next, MTT assay was performed. All experiments were performed in triplicate.

### *In vivo* oxygen generation capability of UP

Female BALB/c mice were inoculated with 100 μL of 1×10^6^ cells/mL 4T1 cells on the right side of the back to establish a subcutaneous murine TNBC model on Day 0. After tumor volumes reach ~ 400 mm^3^, the mice were randomly divided into 3 groups (3 mice for each) and treated with 1) saline (NS), 2) NOCC-AHA, or 3) UP. The dose of αPD-1 was 40 μg/mouse, and the dose of CAT was 1 mg/mouse. The saline and hydrogels were intratumorally injected, and the injection volume was 100 μL. Photoacoustic (PA) imaging was performed to detect the saturated tumor oxygen before and 2 h after injection through a multispectral optoacoustic tomography (MSOT) imaging system, inVision 128, iThera Medical, Germany 28. The PA signals of saturated tumor oxygenation were quantitatively analyzed by MSOT software.

### *In vivo* antitumor efficacy

Subcutaneous murine TNBC models were established as previously described. When tumor sizes were 200~300 mm^3^, the mice were randomly assigned to 6 groups (5 mice for each) and treated with 1) saline (NS), 2) NS+2 Gy, 3) NOCC-AHA, 4) NOCC-AHA+2 Gy, 5) UP, or 6) UP+2 Gy. The doses were the same. The saline and hydrogels were intratumorally injected. 24 h later, the mice were anesthetized, and treated with 2 Gy RT as previously reported 29. Tumor volumes (calculated by the following equation: volume = 0.5 × tumor length × tumor width^2^) and body weights were measured and recorded every 2 days. Blood samples were collected after observation. Main organs were collected for hematoxylin and eosin (H&E) staining, and tumors for H&E staining, Ki-67 staining, and TUNEL assays. In another experiment, the survival rate was observed.

### Analysis of the abscopal effect

Subcutaneous murine TNBC models were established as previously described. On Day 4, distant tumors were inoculated on the left side. After another 5 days, the mice were randomly assigned to 4 groups (5 mice for each) and received treatments on the right tumors: 1) NS, 2) NS+2 Gy, 3) UP, and 4) UP+2 Gy. The doses were the same. The saline and hydrogels were intratumorally injected. 24 h later, RT was performed on the right tumors as previously reported 29. Tumor sizes on both sides were recorded every 2 days. The distant tumors were collected for further tests. In another experiment, the survival rate was observed.

### Assessment of immune memory

Immune memory was assessed in the rechallenge models. Subcutaneous murine TNBC models were established as previously described on Day 0. After tumor volumes reached ~ 200 mm^3^, the mice were divided into 4 groups (5 mice for each) and treated with 1) NS, 2) NS+2 Gy, 3) UP, or 4) UP+2 Gy. The doses were the same. The saline and hydrogels were intratumorally injected. 24 h later, RT was performed as previously reported 29. 4 days later, the first tumors were removed completely by surgery. After another 14 days, the second tumors were inoculated on the other side of the back. The second tumor volumes were recorded every 2 days. In another experiment, the survival mice were observed.

### Antitumor immune responses *in vivo*

Immune responses were detected with the abovementioned three models. In brief, 3 days after RT, or on the day of tumor rechallenge, the tumors, spleens, lymph nodes, or blood samples were collected. IFN-γ levels in blood samples were tested by ELISA. Tumors, spleens, and lymph nodes were cutted, collagenase digested, blood cell lysed, grinded, infiltrated, centrifugated and resuspended to prepare single-cell suspensions. Afterward, the cell suspensions were stained with appropriate antibodies, and then analyzed by flow cytometry (FCM), ACEA, USA. In unilateral model, T cells (CD4^+^, CD8^+^), activated T cells (CD4^+^CD69^+^, CD8^+^CD69^+^), and Treg cells in tumors were analyzed. T cells (CD4^+^, CD8^+^) in spleen and DCs (CD80^+^/CD86^+^CD11c^+^) in lymph nodes were tested. In distant tumor model, T cells (CD4^+^, CD8^+^) in distant tumors and spleens were detected. Effector memory T cells (T_EM_) in spleen were dectected in rechallenged model.

### Statistical analysis

Statistical analysis was carried out by Prism 9, USA. The results are noted as the mean ± SD, and analyzed by one-way analysis of variance (ANOVA). Survival curves were based on the Kaplan‒Meier method. Statistical significances were determined by Mann‒Whitney U tests. A *p* value < 0.05 on a 2-tailed test was considered to indicate statistical significance. * p < 0.05, ** p < 0.01, *** p < 0.001, **** p < 0.0001.

## Results

### Preparation and characterization of UP

**Figure 2:**
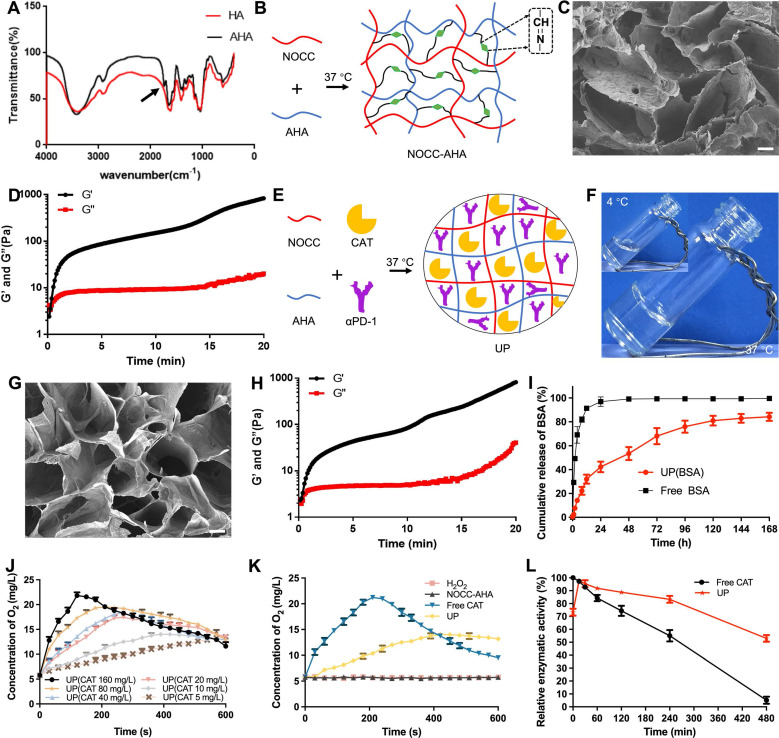
Preparation and characterization of UP. A, FITR spectra. The arrow indicates the characteristic absorption peak of the aldehyde group. B, Illustration of NOCC-AHA hydrogel preparation. C, SEM image of NOCC-AHA. Scale bar, 200 μm. D, G' and G'' of NOCC-AHA. E, Illustration of UP preparation. F, Images of UP at 4 °C (inserted) and 37 °C. G, SEM image of UP. Scale bar, 200 μm. H, G' and G'' of UP. I, Drug release behavior of UP. J, Oxygen generation capability of UP with different CAT loadings. K, Oxygen generation capability of UP compared with that of free CAT. L, Relative enzymatic activity of UP and free CAT.

AHA was synthesized successfully according to the ^1^H-NMR (Figure S1 and S2) and FTIR (Figure 2A) results. The oxidation rate of AHA was determined to be 28.13 ± 4.69% by NH_2_OH·HCl method. The obtained AHA was then applied to prepare a hydrogel named NOCC-AHA through a cross-linking reaction between the amino group of NOCC and the aldehyde group of AHA at 37 °C, as shown in Figure 2B. The NOCC-AHA hydrogel exhibited a porous structure (Figure 2C), which facilitated the movement of the loaded drugs, and a gelation time of 21 s from 4 °C to 37 °C (Figure 2D and S3), which indicated that NOCC-AHA could quickly form a hydrogel at body temperature. We further analyzed the cytotoxicity of NOCC-AHA to 3T3 cells and 4T1 cells. The results shown in Figures S4 and S5 revealed that NOCC-AHA was not toxic to either cell line. These results suggested that the obtained NOCC-AHA was suitable for delivering drugs *in vivo* as a hydrogel.

The preparation of UP is illustrated in Figure 2E. As shown in Figure 2F, UP converted quickly from sol (4 °C) to gel (37 °C), indicating that the loaded cargoes did not affect the formation of hydrogel. SEM revealed that the formed UP hydrogel displayed a porous structure (Figure 2G), suggesting that the hydrogel structure of NOCC-AHA could not be broken by the addition of CAT and αPD-1. Rheological measurements of UP were shown in Figure 2H. The gelation time was within 61 s, which was much longer than that of NOCC-AHA and was preferable in practice since it provided enough time for injection. We then analyzed the cytotoxicity of UP with respect to 3T3 cells and 4T1 cells. In Figure S6 and S7, no significant difference was detected in both NIH3T3 and 4T1 cells at 24 h and 48 h after UP treatment, indicating that UP was not directly cytotoxic to these two cell types. These results suggested that our injectable UP was successfully prepared.

The drug release behavior of UP was analyzed by a dialysis method. The drugs loaded in UP were all proteins (CAT and αPD-1). Here, we assessed the release behavior of protein from UP using the classical standard protein BSA as a substitute because of its high stability. As shown in Figure 2I, more than 90% of the BSA was detected within 12 h in the free BSA group, in which the cumulative release quickly balanced after 24 h, suggesting the rapid BSA release from the free BSA solution. In contrast, UP released only approximately 31.49% of the BSA in 8 h and gradually became balanced after 120 h, with a cumulative release of approximately 81.00%, indicating a much slower release behavior of protein from UP. These results suggested that UP could be applied as a preferable delivery system to store and slowly release proteins such as CAT and αPD-1.

To assess the ability of UP to circumvent the limitation of hypoxia after RT by generating oxygen* in situ* through the delivery of CAT, the catalytic activity of UP was analyzed *in vitro*. As shown in Figure 2J, when adding UP1 mmol/L H_2_O_2_ solutions, the higher the added CAT concentration of UP , the faster the initial oxygen generation. However, the faster oxygen was generated, the faster H_2_O_2_ was consumed. With reaction time, the generation of oxygen decreased gradually. This phenomenon was due to the initial burst release of CAT from UP, which could lead to the rapid consumption of substrates such as H_2_O_2_ at the tumor site. The higher the concentration was, the more obvious the initial burst release was. When the CAT concentration in the UP was 10 mg/L, oxygen generation was relatively stable compared with that in the UP with more than 10 mg/L CAT and clearly more efficient than that in the UP with 5 mg/L CAT. The generation of oxygen in H_2_O_2_ solution by UP was more stable than that by free CAT (Figure 2K), indicating that, as a hydrogel delivery system, UP could supplement oxygen stably due to the slow release of CAT. Considering the existence of proteases at the tumor site, we further assess the *in vitro* relative enzymatic activity of UP in the presence of Proteinase K. As displayed in Figure 2L, the relative enzymatic activity of free CAT decreased rapidly and remained stable at only 5.16 ± 2.76% in 480 min. However, the relative enzymatic activity of UP increased quickly (reaching 97.67 ± 1.52%) in the first 15 min but then gradually decreased to 52.95 ± 2.63% in 480 min. This result suggested that UP, as a hydrogel delivery system, could significantly decrease the inactivation of UP caused by proteases and efficiently maintain the catalytic activity of UP in the presence of proteases.

### Antitumor effects *in vivo*

Before evaluating the *in vivo* antitumor effect, the oxygen generation capability of UP was assessed through PA imaging. As shown in Figure S8A, no obvious differences in the PA signals of saturated oxygen were observed between the NS and NOCC-AHA groups before and 2 h post injection. However, in the UP group, obviously stronger PA signals of saturated oxygen at tumor sites were observed at 2 h after injection than before injection. Saturated oxygen in tumors with UP treatment was significantly greater at 2 h after injection than before injection (Figure S8B). These results indicated that UP could effectively generate oxygen at tumor sites.

**Figure 3:**
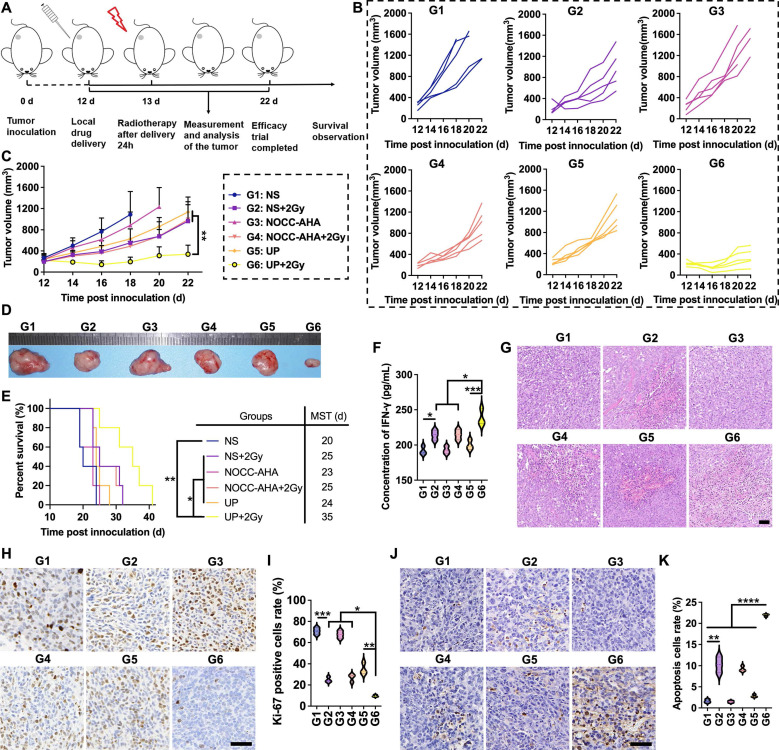
Antitumor efficacy* in vivo*. A, Illustration of the time axis for the antitumor efficacy experiment. B, Tumor growth of each mouse. C, Average tumor growth curves of each group. n = 5. D, Images of representative *ex vivo* tumors. E, Survival rates of tumor-bearing mice. The MST of each group is listed. n = 5. F, IFN-γ concentrations in serum. G, H&E staining images of tumor tissues of each group. Scale bar, 50 μm. H, Ki-67 staining images of tumor tissues of each group. Scale bar, 50 μm. I, statistical results of H. J, TUNEL staining images of tumor tissues of each group. Scale bar, 50 μm. K, statistical results of J.

The *in vivo* antitumor effect of UP combined with RT was analyzed, as illustrated in Figure 3A. After the administration of UP, RT was performed the next day in the UP+2 Gy group. The individual tumor growth in each group is shown in Figure 3B. One mouse in NS group died on Day 19, and two on Day 21. On Day 21, two mice in the NOCC-AHA group died. Thus, the average tumor growth curves of NS and NOCC-AHA groups shown in Figure 3C were stopped on Days 18 and 20, respectively. Among the other 4 groups, the tumor growth in the UP+2 Gy group was significantly slower than those in other groups, including the NS+2 Gy and NOCC-AHA+2 Gy groups. No obvious difference was observed between the NS and NOCC-AHA groups or between the NS+2 Gy and NOCC-AHA+2 Gy groups, suggesting that NOCC-AHA had no obvious antitumor effect. When the efficacy trial ended on Day 22, the tumors in the UP+2 Gy group were obviously smaller than those in the other groups were (Figure 3D). Furthermore, tumor-bearing mice in UP+2 Gy group with a median survival time (MST) of 35 days had the longest survival time, comparing with those in NS+2 Gy (MST of 25 days), NOCC-AHA+2 Gy (MST of 25 days), and UP (MST of 24 days) groups (Figure 3E).

The IFN-γ concentration in the blood was subsequently detected. As shown in Figure 3F, the IFN-γ concentration in the NS+2 Gy group (214.1 ± 7.03 pg/mL) was significantly greater than that in the NS group (191.9 ± 6.0 pg/mL), indicating that 2 Gy of radiation could increase the secretion of IFN-γ to enhance the antitumor effect. However, the IFN-γ concentration in the NS+2 Gy and NOCC-AHA+2 Gy groups (214.7 ± 7.2 pg/mL) was still lower than that in the UP+2 Gy group (238.3 ± 11.8 pg/mL), which was the highest of all the groups. These results were in line with those of the tumor growth curve and survival time. Meanwhile, as shown in Figure 3G, obviously more nuclear shrinkage and fewer tumor cells were observed in the UP+2 Gy group than in the NS+2 Gy, NOCC-AHA+2 Gy, and UP groups. Ki-67 staining, as shown in Figure 3H and 3I, revealed significantly fewer Ki-67-positive cells in the NS+2 Gy group (24.90 ± 2.55%) than in the NS group (70.84 ± 3.28%), and the lowest level of Ki-67 was detected in the UP+2 Gy group (9.63 ± 0.94%). TUNEL staining, as shown in Figure 3J and 3K, revealed similar results: although the NS+2 Gy group (10.20 ± 1.85%) exhibited a much higher level of apoptosis than the NS group did (1.63 ± 0.41%), the UP+2 Gy group (21.92 ± 0.29%) still exhibited a significantly higher level of apoptosis than the NS+2 Gy group and all the other groups. These results were in accordance with the results of the efficacy trial and IFN-γ concentration in blood, suggesting that UP could significantly enhance the antitumor effect of RT.

### Antitumor immune responses

**Figure 4:**
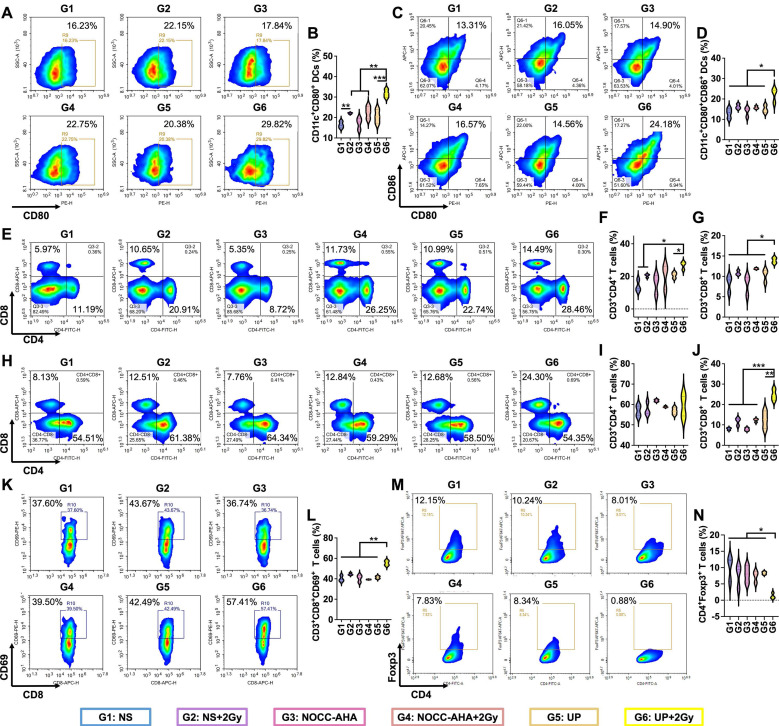
Systemic antitumor immune responses. FCM analysis (A) and statistical results (B) of CD80^+^ in CD11c^+^ DCs. FCM analysis (C) and statistical results (D) of CD80^+^CD86^+^ in CD11c^+^ DCs. FCM analysis (E) and statistical results for CD4^+^ (F) and CD8^+^ (G) in CD3^+^ T cells in spleen. FCM analysis (H) and statistical results for CD4^+^ (I) and CD8^+^ (J) in CD3^+^ T cells in tumors. FCM analysis (K) and statistical results (L) of CD69^+^ in CD8^+^ T cells in tumors. FCM analysis (M) and statistical results (N) of CD4^+^Foxp3^+^ in CD3^+^ T cells in tumors.

Inspired by the excellent antitumor efficacy, we further analyzed immune responses in tumor-bearing mice. DCs in lymph nodes on the treated side were first analyzed. In Figure 4A and 4B, the highest rate of CD11c^+^CD80^+^ DCs was detected in the UP+2 Gy group. Although no obvious difference in CD11c^+^CD86^+^ DCs was observed between the UP+2 Gy group and the NS+2 Gy, NOCC-AHA+2 Gy, or UP groups (Figure S9), a significantly greater proportion of CD11c^+^CD80^+^CD86^+^ DCs was detected in the UP+2 Gy group than in the other groups, including the NS+2 Gy, NOCC-AHA+2 Gy, and UP groups (Figure 4C and 4D). These results indicated that UP+2 Gy could effectively promote the maturation of DCs in lymph nodes to induce antitumor immune responses. T cells in spleen were subsequently analyzed in Figure 4E, 4F, and 4G. The highest populations of CD3^+^CD4^+^ and CD3^+^CD8^+^ T cells were detected in the UP+2 Gy group, which were significantly greater than those in the NS+2 Gy and UP groups. These results indicated that, in combination with RT, UP could effectively induce systemic antitumor immune responses.

We next analyzed the T cells in tumors to evaluate the outcomes of the induced systemic antitumor immune responses. As shown in Figure 4H, 4I, and 4J, the UP+2 Gy group presented the highest number of CD3^+^CD8^+^ T cells of all groups, including NS+2 Gy, NOCC-AHA+2 Gy, and UP groups, although no obvious difference was noted in CD3^+^CD4^+^ T cells. Similarly, there was no difference in CD3^+^CD4^+^CD69^+^ T cells (Figure S10), but a significantly greater population of CD3^+^CD8^+^CD69^+^ T cells was detected in UP+2 Gy group than in other groups (Figure 4K and 4L), indicating that significantly more cytotoxic T lymphocytes (CTLs) had been activated after UP and RT treatment. Furthermore, the lowest rate of Treg cells (CD3^+^CD4^+^Foxp3^+^) was observed in the UP+2 Gy group (Figure 4M and 4N), suggesting that significantly fewer Treg cells remained in tumor tissues. These results indicated that UP could effectively enhance the systemic antitumor immune responses of RT by increasing CTL activation and decreasing Treg cell infiltration.

### Abscopal effect assessment

**Figure 5:**
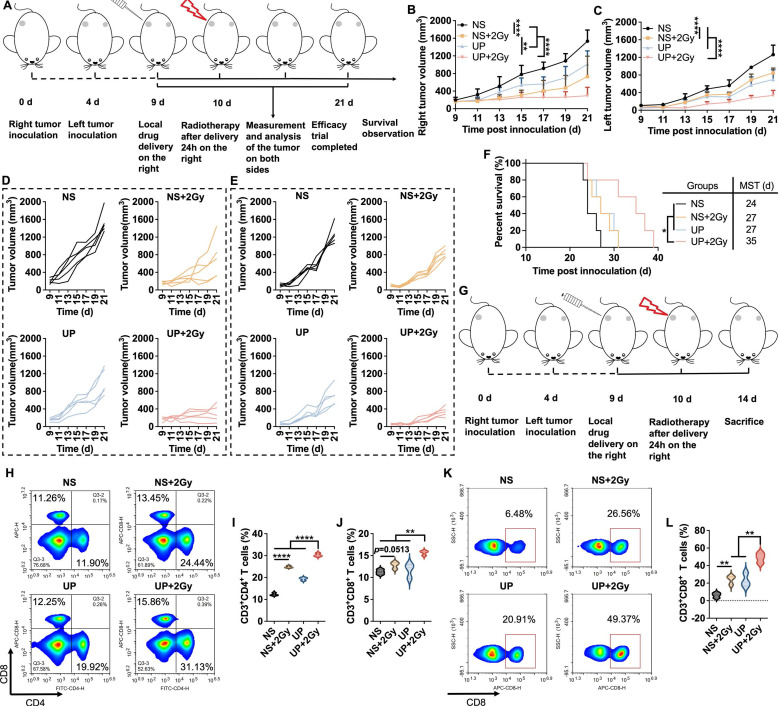
Abscopal effect. A, Illustration of the time axis for the abscopal efficacy evaluation experiment. B, Average growth curves of the right (treated) tumors. n = 5. C, Average growth curves of the left (distant) tumors. n = 5. D, Growth curves of right (treated) tumors of each mouse in each group. n = 5. E, Growth curves of the left (distant) tumors of each mouse in each group. n = 5. F, Survival rates. The MST of each group is listed. n = 5. G, Illustration of the time axis for the abscopal effect detection experiment. FCM analysis (H) and statistical results for CD4^+^ (I) and CD8^+^ (J) in CD3^+^ T cells in spleen. FCM analysis (K) and statistical results (L) of CD8^+^ in CD3^+^ T cells in tumors.

The abscopal effect of UP and RT combination therapy was assessed in a bilateral TNBC mouse model. The experiments were performed according to the time axis illustrated in Figure 5A. In Figure 5B and 5D, compared with those in all the other groups, the right (treated) tumors in the UP+2 Gy group exhibited the slowest growth, which was in accordance with the results of the unilateral TNBC models. As shown in Figure 5C and 5E, the left (distant) tumors in the NS+2 Gy group displayed significantly slower growth than those in the NS group did. However, the growth of the distant tumors in NS+2 Gy group was still notably faster than that in UP+2 Gy group, which exhibited the slowest tumor growth. Similar results were observed for the survival of mice, as shown in Figure 5F. Compared with those in the NS (MST of 24 days), NS+2 Gy (MST of 27 days), and UP (MST of 27 days) groups, tumor-bearing mice in the UP+2 Gy group clearly survived longer. These results suggested that UP and RT combination therapy inhibited the growth of distant tumors and prolonged the survival time of mice effectively, resulting in a strong abscopal effect.

We next analyzed the T cells in the spleens and distant tumors to assess the abscopal effect following the experimental time axis shown in Figure 5G. As shown in Figure 5H, 5I, and 5J, in the spleen, significantly greater populations of CD3^+^CD4^+^ and CD3^+^CD8^+^ T cells were detected in UP+2 Gy group than in other groups, including NS+2 Gy group, further indicating that UP promoted the systemic antitumor immune response to RT. In distant tumors, although no obvious difference was observed in CD3^+^CD4^+^ T cells (Figure S11), the rate of CD3^+^CD8^+^ T cells in NS+2 Gy group was much greater than that in NS group but significantly lower than that in UP+2 Gy group, which was the highest of all (Figure 5K and 5L). These results further revealed that, in addition to systemic antitumor immune responses, UP combined with RT had an effective abscopal effect on distant tumors.

### Assessment of immune memory effects

**Figure 6:**
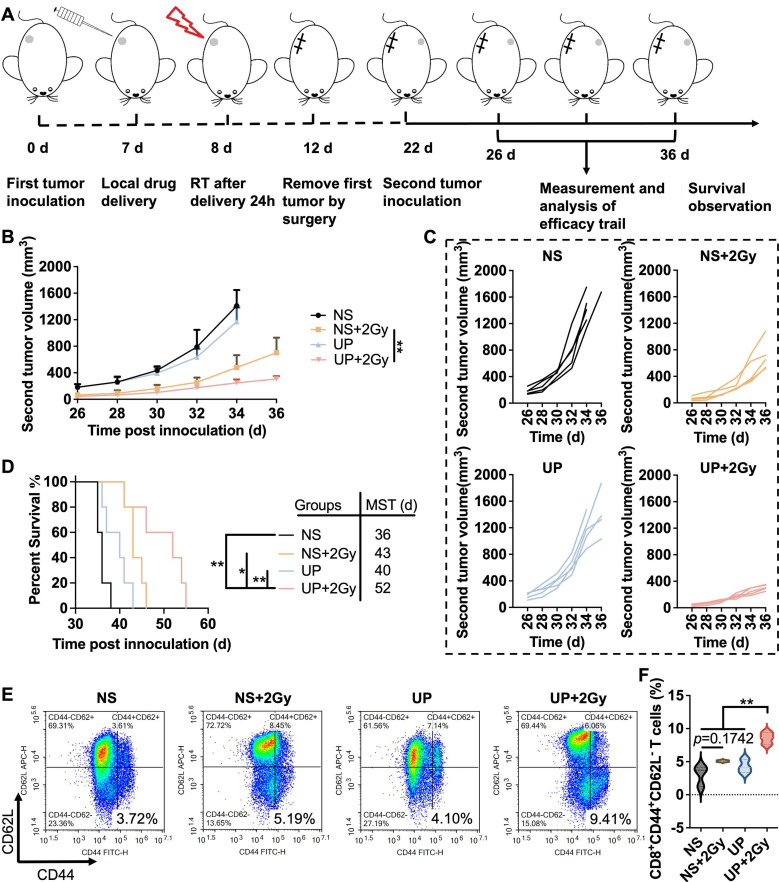
Immune memory effect. A, Illustration of the time axis for the immune memory efficacy evaluation experiment. B, Average growth curves of the second (rechallenged) tumors. n = 5. C, Growth curves of the second (rechallenged) tumors of each mouse in each group. n = 5. D, Survival rates. The MST of each group is listed. n = 5. FCM analysis (E) and statistical results (F) of CD44^+^CD62L^-^ in CD3^+^CD8^+^ T cells in spleen.

The assessment of the immune memory effect was conducted following the time axis shown in Figure 6A. The first tumor was treated with UP on Day 7, given 2 Gy of radiation the following day, and removed completely by surgery. 14 days after RT, the second tumors were inoculated. The growth of the second tumors was recorded. As shown in Figure 6B and 6C, the growth of second tumors in UP+2 Gy group was the slowest. On Day 35, two tumor-bearing mice died in the NS group, and one died in the UP group. Another two died in NS, and one died in UP on Day 36. Among the other two groups, the second tumor growth of UP+2 Gy group was significantly slower than that of NS+2 Gy group, indicating that UP could effectively promote the immune memory effect of RT to inhibit rechallenged tumor growth. Moreover, tumor-bearing mice in the UP+2 Gy group (MST of 52 days) had longer survival times than those in the NS (MST of 36 days), NS+2 Gy (MST of 43 days), and UP (MST of 40 days) groups did (Figure 6D), suggesting that the immune memory-promoting effect of the UP and RT combination could effectively prolong the survival time of mice. Furthermore, T_EM_ cells in spleen were analyzed. As shown in Figure 6E and 6F, no obvious difference in CD3^+^CD8^+^CD44^+^CD62L^-^ T cells was detected between NS and NS+2 Gy groups, suggesting that RT alone could not induce an effective immune memory effect. The proportion of CD3^+^CD8^+^CD44^+^CD62L^-^ T cells in UP+2 Gy group was greater than that in NS, NS+2 Gy, and UP groups, indicating that UP could effectively promote the immune memory effect that RT alone could not arouse. These results confirmed that, in addition to promote systemic antitumor immune responses, UP and RT combination promoted effective immune memory effects.

### *In vivo* toxicity assessment

During all the animal experiments, no obvious abnormal behavior was observed in the tumor-bearing mice. No marked side effects on the appearance (hair, tail, eyes, etc.) of tumor-bearing mice were noted. The body weights were recorded for all three groups. As shown in Figures S12-S14, there was no obvious difference in the average body weights. Furthermore, H&E staining of the heart, livers, spleen, lungs, and kidneys was performed. In Figure S15, the tissue structures of the heart, livers, spleens, lungs, and kidneys were all clear. No obvious difference in the sizes, shapes, or arrangement of cells was observed. No obvious signs of inflammation or necrosis in these organs were detected. These results suggested that UP and RT combination displayed no or low toxicity *in vivo*, and no distinct harm was detected in any of the tumor-bearing mice treated with UP.

## Conclusion and Discussion

In this study, we aimed to improve the antitumor immunity of RT through circumventing the limitations and capitalizing on the strength of RT. Briefly, NOCC-AHA hydrogels with low toxicity, degradability, and injectivity were first prepared. UP was subsequently prepared by coloading αPD-1 and CAT in the NOCC-AHA hydrogel. UP can be directly injected into local tumors and can be conveniently administered, slowly release loaded drugs, and improve the stability and physiological activity of loaded proteins under physiological conditions. The *in vivo* efficacy of UP combined with RT was subsequently analyzed. In subcutaneous TNBC unilateral models, bilateral models, and rechallenge models, UP combined with RT showed excellent antitumor efficacy and safety, significantly inhibiting the growth of primary, distant, and rechallenged tumors and prolonging survival time. Furthermore, the results also revealed that UP could significantly increase systemic antitumor immune responses, improve abscopal effect, and promote immune memory effect of RT. In conclusion, UP successfully increased antitumor immunity with robust abscopal effects and durable immune memory effects through the strategy of circumventing limitations and capitalizing strength. Our strategy of UP and RT combination therapy provides new ideas and approaches for TNBC treatment and RT enhancement.

Recently, hydrogels have emerged as transformative platforms in oncology, significantly advancing the application of radiotherapy and immunotherapy by enabling localized, sustained, and stimuli-responsive drug delivery 33,34. Their biocompatible, highly tunable nature allows them to function as versatile depots that can be injected or implanted directly at tumor sites, such as post-resection cavities, to maximize therapeutic efficacy while minimizing systemic toxicity 33. In radiotherapy, hydrogels enhance treatment by mitigating side effects and overcoming radioresistance 30,33. In immunotherapy, hydrogels excel as localized immunomodulatory niches that reprogram the immunosuppressive tumor microenvironment 31,34. They facilitate the controlled release of immune checkpoint inhibitor (such as αPD-1) cytokines or adjuvants, avoiding adverse systemic immune-related effects while promoting robust antitumor responses 34. Their ability to reverse immunosuppression by alleviating hypoxia, repolarizing macrophages, or recruiting T cells makes them ideal for synergizing with immunotherapy 27,31,33,34. Most notably, hydrogels integrate radiotherapy and immunotherapy by harnessing radiation-induced immunogenic cell death to release tumor antigens while simultaneously releasing checkpoint inhibitors to unleash a potent, systemic antitumor immune response 33,34. The combination of RT with the UP hydrogel developed in this study successfully and significantly potentiated the efficacy of radiotherapy and immunotherapy combination therapy and promises to overcome treatment resistance in solid tumors.

## Supplementary Material

Supplementary figures.
