# Supplementary material for: Limitation-circumventing and strength-capitalizing hydrogel potentiates durable antitumor immunity and robust abscopal effect in radiotherapy
Source: Theranostics. 2026 Jan 1;16(3):1432–44. doi: 10.7150/thno.118974 (PMC12679582; doi:10.7150/thno.118974)
Supplement: Supplementary file 1 — Supplementary figures. [file thnov16p1432s1.pdf]

Supporting Information for

Limitation-circumventing strength-capitalizing hydrogel potentiates durable antitumor immunity and robust abscopal effect in radiotherapy

Haijun Li[1,2,+], Xianzhou Huang[1,+], Jin Yang[1,+], Liping Bai[1], Meiling Shen[1], Yaqin Zhao[3,4], Changyang Gong[1], Yanjie You[5,6,*], Qinjie Wu[1,*]

[1] Department of Biotherapy, Cancer Center and State Key Laboratory of Biotherapy, West China Hospital, Sichuan University, Chengdu, 610041, P. R. China

[2] Department 4 of Oncology, Cancer Center, The Second People's Hospital of Neijiang, Neijaing, 641000, P.R. China

[3] Abdominal Oncology Ward, Cancer Center, West China Hospital, Sichuan University, Chengdu, 610041, P. R. China

[4] State Key Laboratory of Biological Therapy, West China Hospital, Sichuan University, Chengdu, 610041, P. R. China

[5] Department of Gastroenterology, People's Hospital of Ningxia Hui Autonomous Region, Ningxia Medical University, Yinchuan, 750002, P.R. China

[6] Department of Gastroenterology, the Third Clinical Medical College of Ningxia Medical University, Yinchuan, 750002, P.R. China

[*] To whom correspondence should be addressed (Y You and Q Wu). E-mail: youyanjie@163.com and cellwqj@163.com.

[+] These authors contributed equally to this work.

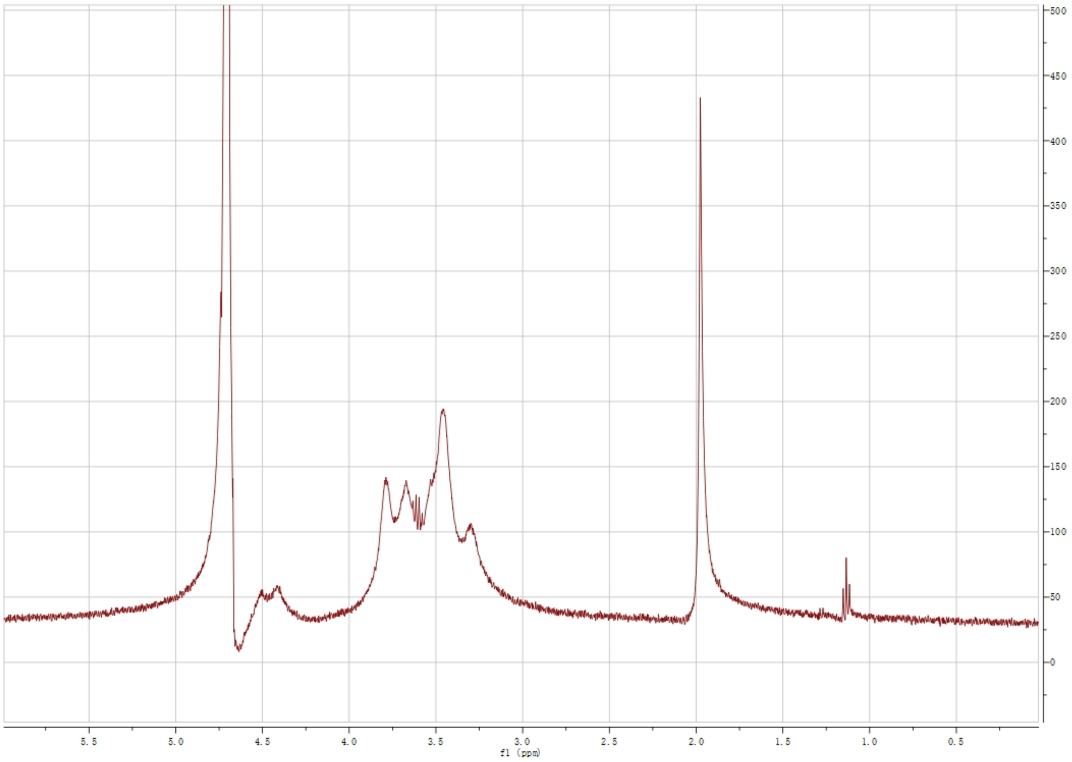

Figure S1. ^{1}H-NMR result of HA

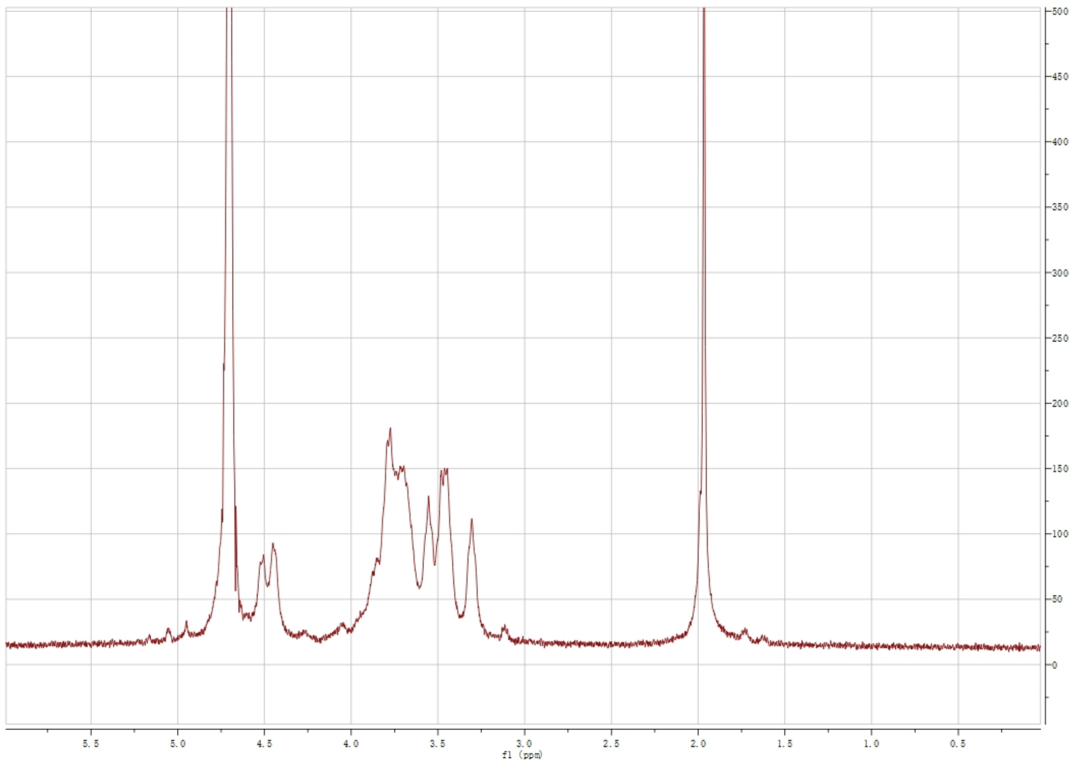

Figure S2. ^{1}H-NMR result of AHA

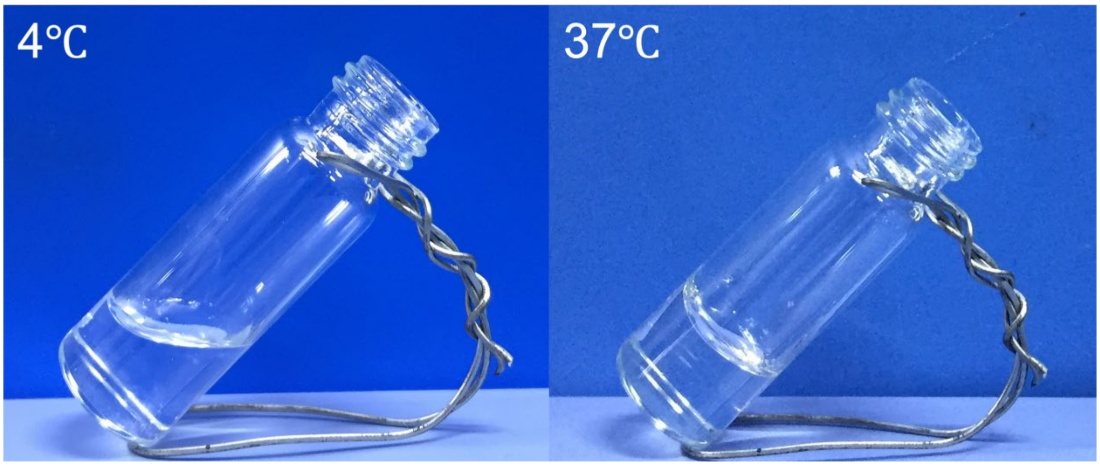

Figure S3. Sol-gel transition of NOCC-AHA from 4℃ to 37℃

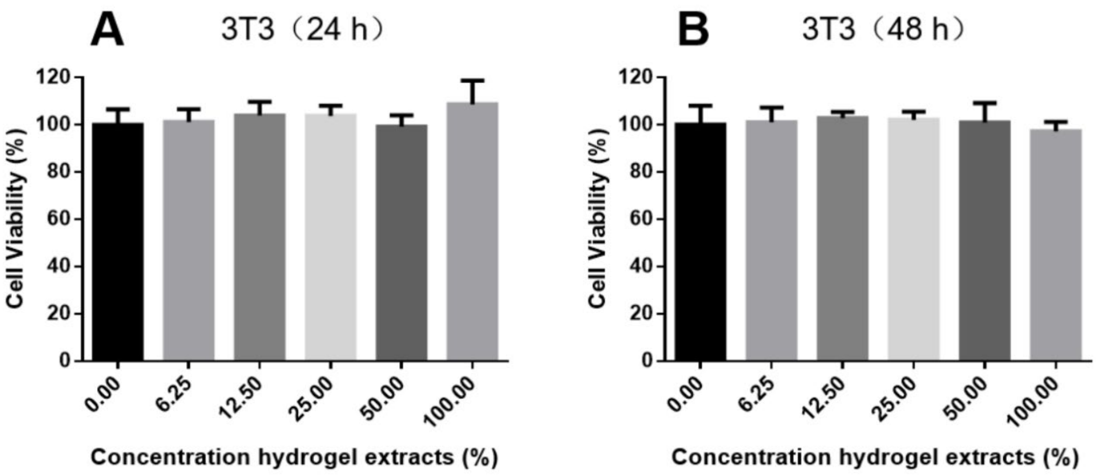

Figure S4. MTT assay of NOCC-AHA on 3T3 cells

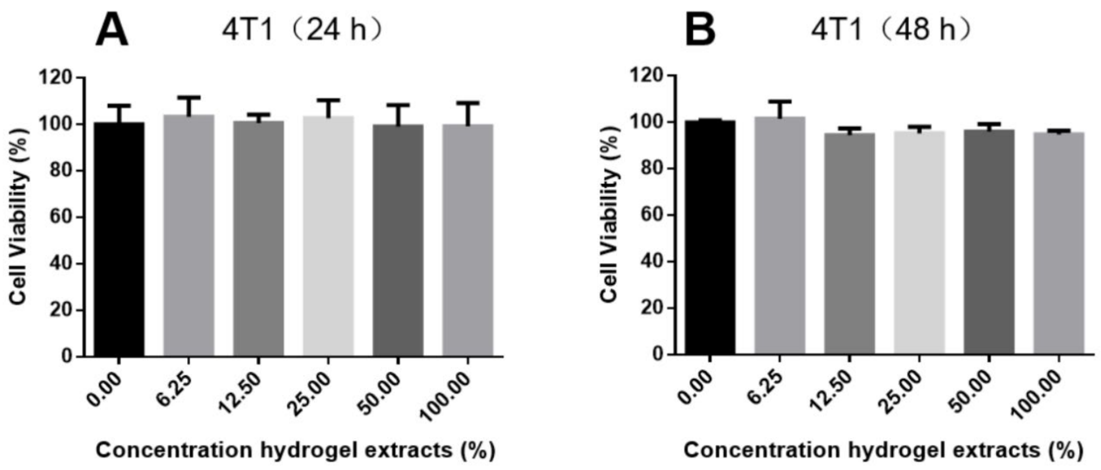

Figure S5. MTT assay of NOCC-AHA on 4T1 cells

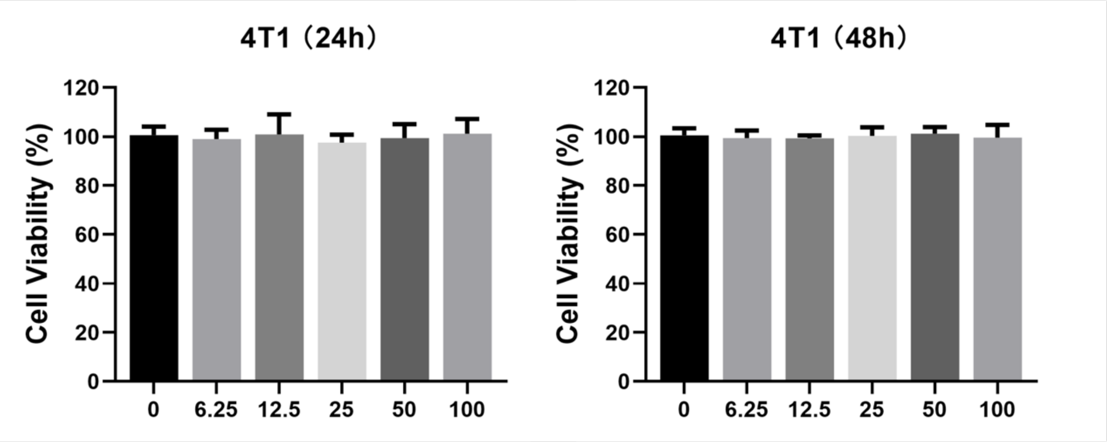

Figure S6. MTT assay of UP on 4T1 cells

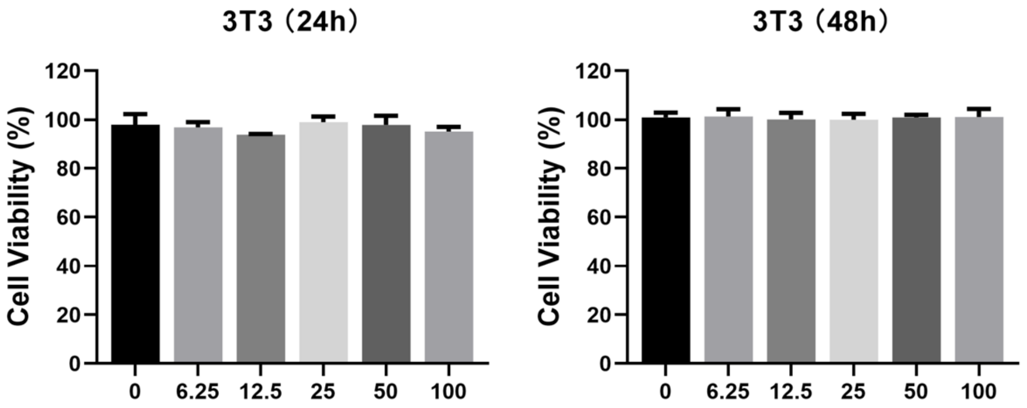

Figure S7. MTT assay of UP on 3T3 cells

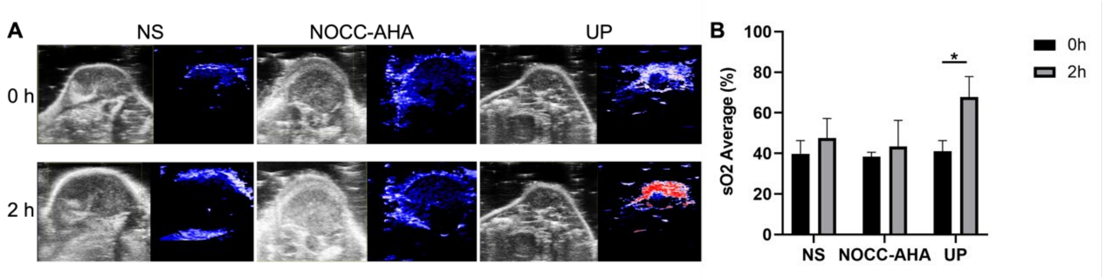

Figure S8. Saturated oxygen in tumors after injection of UP hydrogel determined by PA imaging. A, PA images of saturated oxygen in tumors with different treatments. B, Statistic of saturated oxygen in tumors with different treatments.

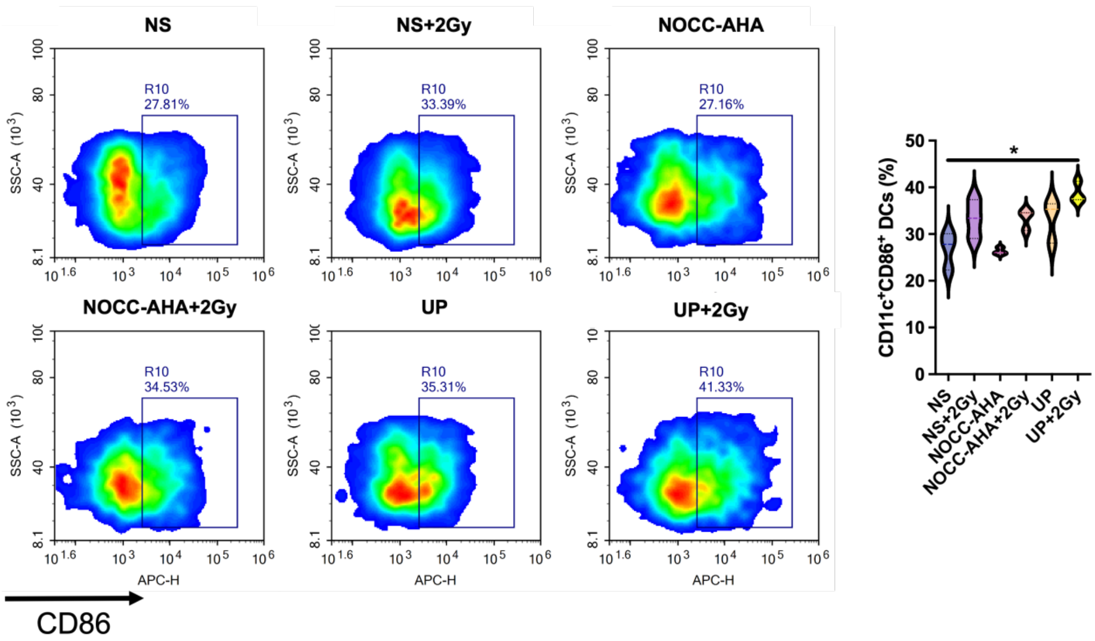

Figure S9. FCM analysis of CD11c$^+$CD86$^+$ DCs in lymph nodes

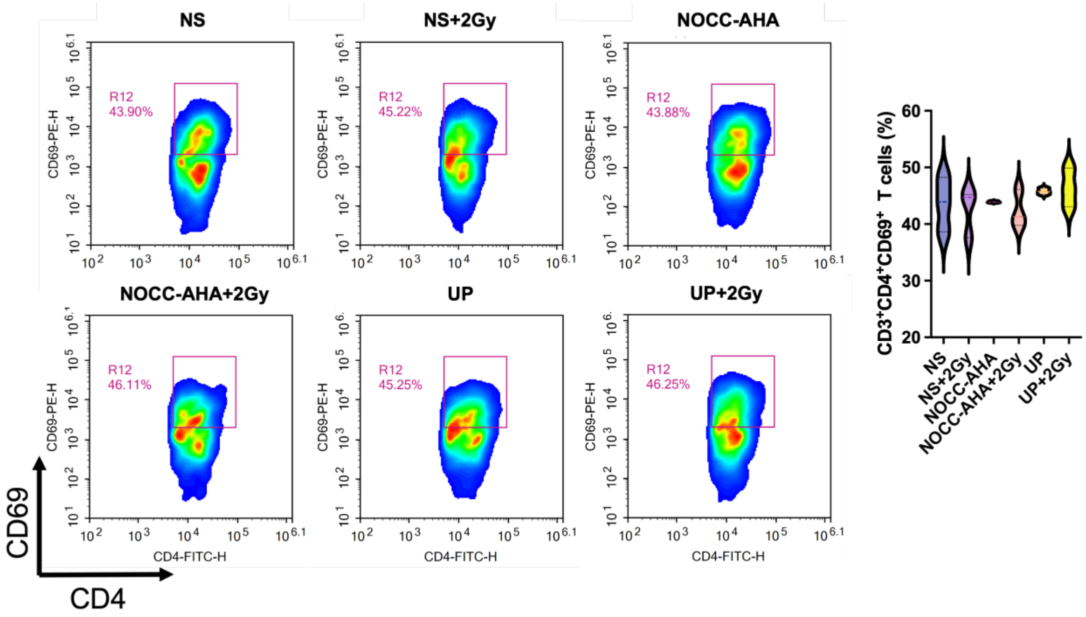

Figure S10. FCM analysis of $CD3^{+}CD4^{+}CD69^{+}$ T cells in tumor tissues

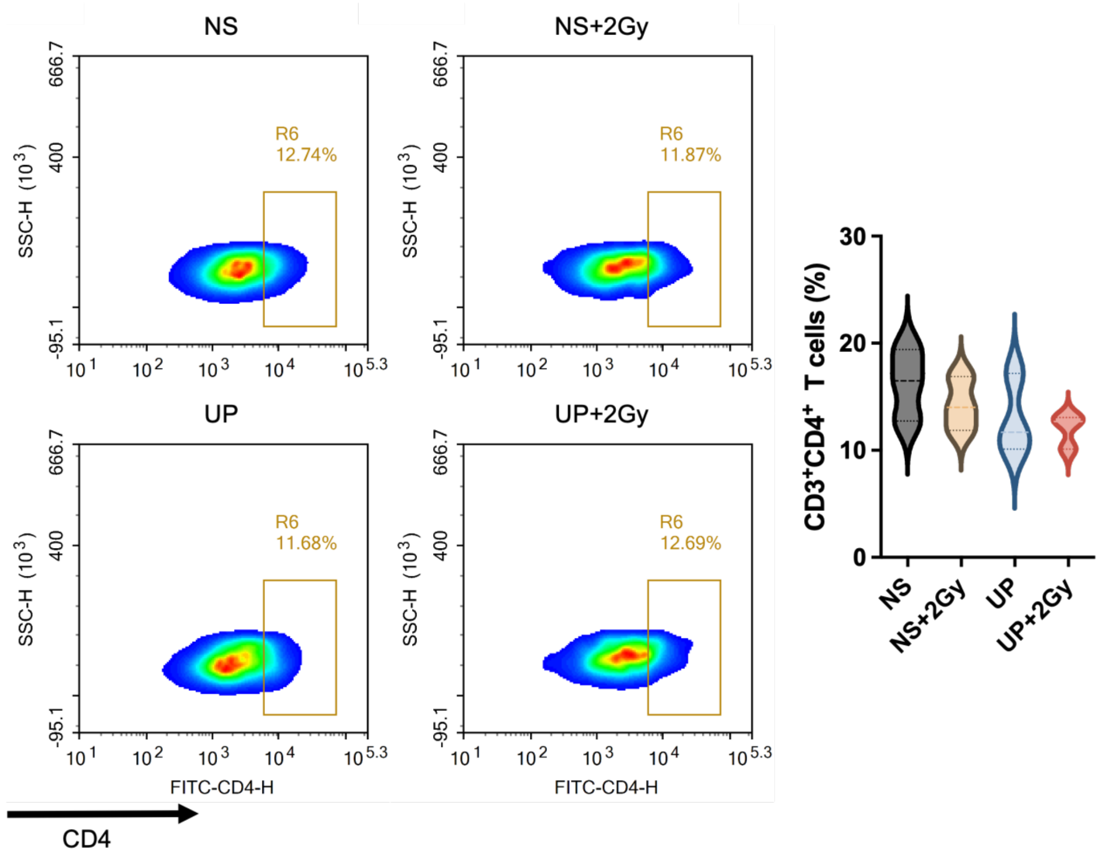

Figure S11. FCM analysis of $CD3^{+}CD4^{+}$ T cells in distant tumor tissues

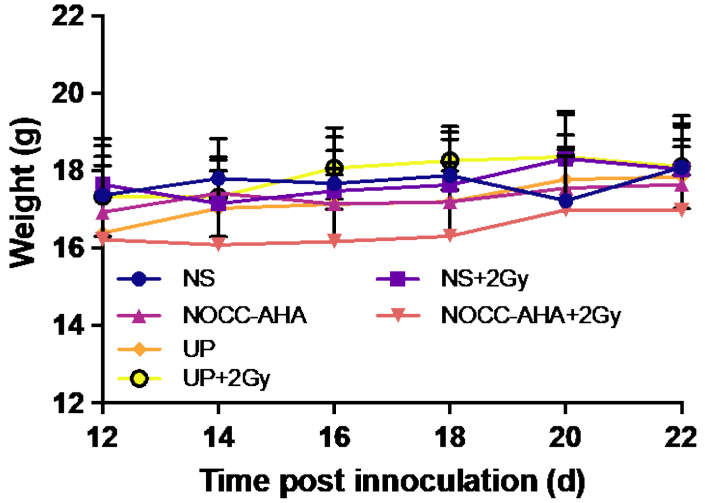

Figure S12. Body weights of tumor-bearing mice in unilateral models

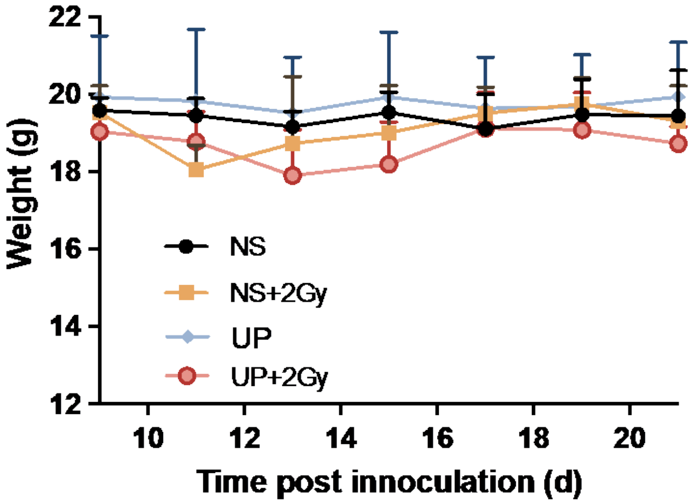

Figure S13. Body weights of tumor-bearing mice in bilateral models

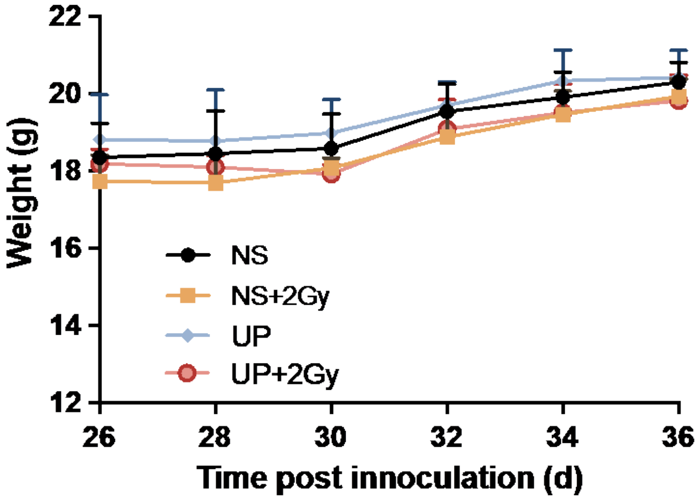

Figure S14. Body weights of tumor-bearing mice in rechallenge models

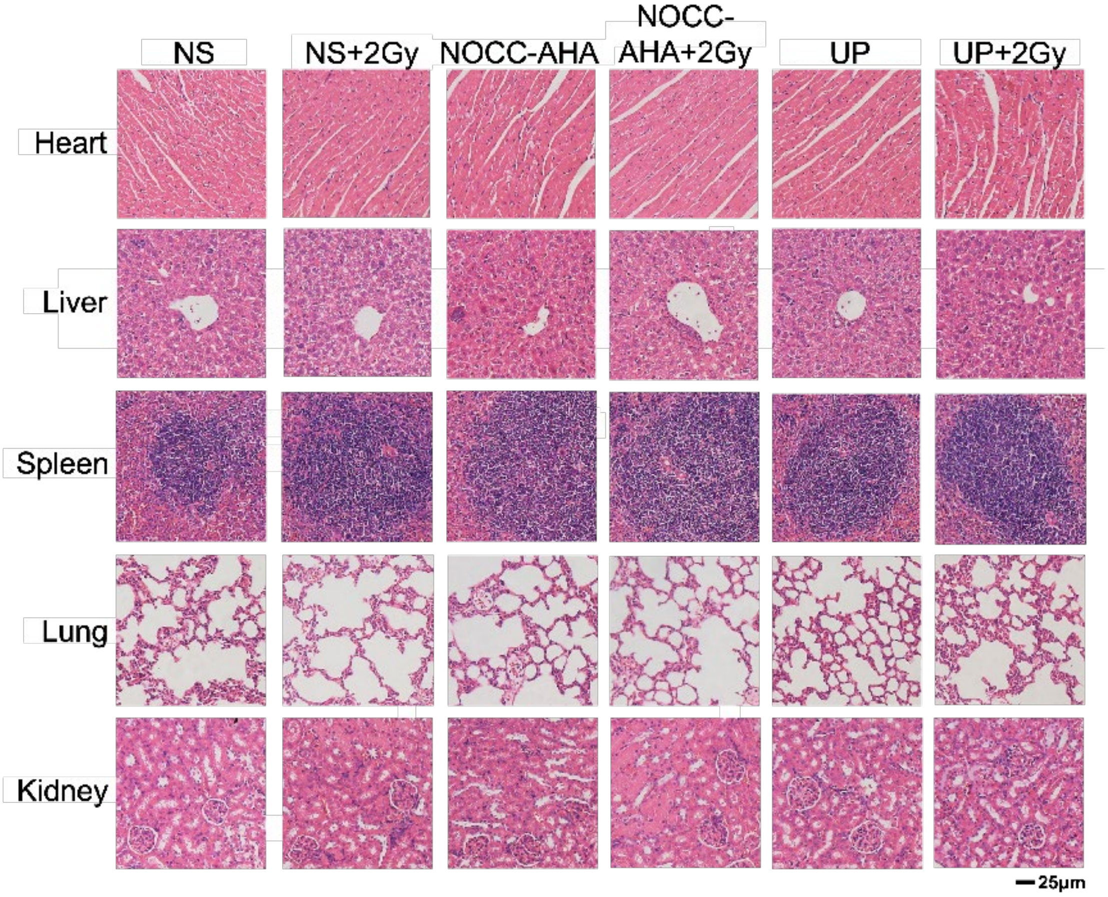

Figure S15. H&E staining of heart, livers, spleen, lungs and kidneys in each group
